# DEPTH2 score was associated with cell proliferation and immune cell infiltrations but not with systemic treatment response in breast cancer

**DOI:** 10.21203/rs.3.rs-5260856/v1

**Published:** 2024-11-20

**Authors:** Kohei Chida, Rongrong Wu, Arya Mariam Roy, Takashi Ishikawa, Kenichi Hakamada, Kazuaki Takabe

**Affiliations:** Roswell Park Comprehensive Cancer Center; Roswell Park Comprehensive Cancer Center; Ohio State University; Tokyo Medical University; Hirosaki University Graduate School of Medicine; Roswell Park Comprehensive Cancer Center

**Keywords:** Breast cancer, Intratumoral genomic heterogeneity, DEPTH2, Gene expression

## Abstract

Intratumoral genomic heterogeneity (ITGH), the existence of genotypic and phenotypic variation within an individual tumor, is known to be a key mechanism in treatment resistance. Deviating gene Expression Profiling Tumor Heterogeneity 2 (DEPTH2) algorithm was developed to estimate ITGH using solely RNA expression data unlike the others that require both DNA- and RNA-expression data. Total of 6,500 breast cancer patients from multiple independent cohorts were analyzed using DEPTH2. High DEPTH2 score patients were associated with worse overall survival consistently across all subtypes in METABRIC, but not in TCGA and SCAN-B cohort. Higher DEPTH2 score was linked to increased cell proliferation, as evidenced by elevated Nottingham histological grades and Ki67 gene expression, as well as enrichment of the cell proliferation-related gene sets, and immune cell infiltrations. DEPTH2 score was significantly higher in triple negative breast cancer among the subtypes but did not reflect with lymph node and distal metastasis. DEPTH2 scores decreased in two but showed no change in another two cohorts after neoadjuvant chemotherapy (NAC). DEPTH2 score was not associated with pathologic complete response after NAC in any subtypes across 3 cohorts. DEPTH2 score may not capture the entire biological aspects of ITGH in breast cancer patients.

## Introduction

Nearly 310,000 women are diagnosed as breast cancer, and over 42,000 succumb to the disease every year in the United States ^1^. The selection of systemic therapy requires consideration of both the clinical and molecular characteristics of the tumor ^2^. However, it is now known that there may be variations in the molecular characteristics among cancer cells within a single tumor. Intratumor genomic heterogeneity (ITGH), which refers to variations in molecular and phenotypic profiles among different cancer cells within a tumor ^3^, represents a significant barrier to effective cancer treatment. This often leads to treatment resistance and worse prognosis. Indeed, our group and others have reported that tumors with high levels of ITGH are associated with reduced immune response and correlated with poorer survival in various cancer types including breast cancer ^4,5^. To this end, methods to evaluate ITGH that predict prognosis and response to treatments are of clinical value. A cautionary point is the terminology “intratumor heterogeneity (ITH)”, which is also used describe the presence of different cell types (such as cancer cells, immune cells, vascular cells, fibroblasts, etc.) within a bulk tumor. While this type of heterogeneity also plays a role in treatment resistance, it is distinct from ITGH, which refers specifically to genetic heterogeneity among cancer cells.

Next-generation sequencing data derived from a “bulk” tumor can reveal subclonal populations. This approach has proven to provide sufficient resolution for assessing a tumor’s overall heterogeneity and could facilitate the study of a larger number of tumors ^6,7^. A number of computational algorithms have been developed to quantify ITGH using whole exome sequencing data, such as Defining ITH based on EntRopy (DITHER) ^8^, PhyloWGS ^9^, Expanding Ploidy and Allele-frequency on Nested Subpopulations (EXPANDS) ^10^, and Mutant-Allele Tumor Heterogeneity (MATH) ^11^. However, these algorithms require both DNA sequencing and RNA-sequence data. Since the vast majority of publicly available mRNA-sequence datasets do not provide linked DNA-sequence data (The Cancer Genome Atlas (TCGA) is an exception), several algorithms were developed to quantify ITGH using RNA-sequence data alone, such as Deviating gene Expression Profiling Tumor Heterogeneity (DEPTH) ^12^ and transcriptome-based ITH (tITH) ^13^. However, both DEPTH and tITH require normal cells as the reference for calculation, which are often not provided in publicly available datasets. Recently, Deviating Gene Expression Profiling Tumor Heterogeneity 2 (DEPTH2), which does not require a normal cell reference was developed, and shown to be comparable to other methods for estimating ITGH ^14^. Although the utility of DEPTH2 was assessed across multiple cancer types, the original report only utilized TCGA cohort ^14^, raising questions about its generalizability and reproducibility. Here, we investigated the clinical relevance of the transcriptome based DEPTH2 in total of 6500 breast cancer patients from 7 independent cohorts.

## Methods

### Clinical data acquisition for breast cancer patients

We analyzed a total of 6,500 breast cancer patient samples that are associated with both clinical and mRNA expression data. These data were retrieved from seven large independent cohorts; The Cancer Genome Atlas (TCGA, *n* = 1077) ^15^, Molecular Taxonomy of Breast Cancer International Consortium (METABRIC, *n* = 1904) ^16^, The Sweden Cancerome Analysis Network - Breast (SCAN-B, *n* = 3069) ^17^, the PROMIX trial (*n* = 275) ^18^, Ramirez et al. (*n* = 61) ^19^, Stickeler et al. (*n* = 57) ^20^, and Parkes et al. (*n* = 57) ^21^. The data were retrieved from cBioPortal (https://www.cbioportal.org) and the Gene Expression Omnibus (GEO) repository of the US National Institutes of Health (https://www.ncbi.nlm.nih.gov/geo), as we have described previously ^22,23^. HER2 overexpression subtype was excluded from the analyses since the observation periods of the cohorts overlapped with the period before and after the introduction of anti-HER2 targeted therapy, which would have a significant effect on outcomes, leading to bias and potentially misleading results. Mutation information was also obtained from cBioportal. We accessed normalized genomic and clinical datasets from the GEO database. Since the TCGA and GEO data are publicly available and do not include identifiable personal information, Institutional Review Board (IRB) approval was not required for our study.

### Statistical analysis

The DEPTH2 algorithm’s R package was accessible at https://github.com/XS-Wang-Lab/DEPTH2, under a GNU GPL open-source license ^14^. The normalized RNA expression matrix (from RNAseq or microarray) of bulk tumor samples was used as input data to calculate the DEPTH2 score. This algorithm estimates ITGH based on the standard deviation of z-scored expression values for a gene set in the tumor and is considered independent of the expression data normalization method. The high and low DEPTH2 score groups were defined by the median. Statistical analyses were performed using R software (version 4.3.2, www.r-project.org). Group comparisons were made using the Mann–Whitney U test and the Kruskal-Wallis test, with Tukey’s boxplots illustrating interquartile ranges. We explored the association between DEPTH2 score and overall survival (OS) outcomes using the Cox proportional hazards regression model, visually represented by Kaplan-Meier survival curves. A *p*-value of less than 0.05 was considered statistically significant in all tests. Correlation analysis was conducted using Spearman’s rank correlation coefficient.

### Gene set enrichment analysis (GSEA)

Functional enrichment analysis of the DEPTH2 score was conducted using GSEA ^24^ on the Hallmark collection from the Molecular Signatures Database ^25^, as we previously reported ^26,27^. Following the Broad Institute’s guidelines, gene sets with a false discovery rate (FDR) of less than 25% were considered to have achieved statistically significant enrichment in the GSEA.

### Cell composition of the tumor microenvironment (TME)

We used the xCell algorithm to investigate tumors with high and low DEPTH2 scores and the presence of stromal and immune cells in the tumor microenvironment (TME) ^28^. Thorsson et al. provided additional scores for insertions and deletions (Indel) and single-nucleotide variant (SNV) neoantigens, silent and non-silent mutations, and homologous recombination defects (HRD)^29^. ITGH calculation was also validated using the ABSOLUTE algorithm which evaluates tumor ploidy estimates representing ITGH based on DNA copy number alteration (CNA) profiles ^30^. Cytolytic activity score (CYT) was calculated using gene expression levels of *perforin and granzyme A* ^31^.

## Results

### High DEPTH2 score was associated with lower overall survival (OS) in METABRIC across all subtypes but not in TCGA and SCAN-B cohorts

**Figure 1:**
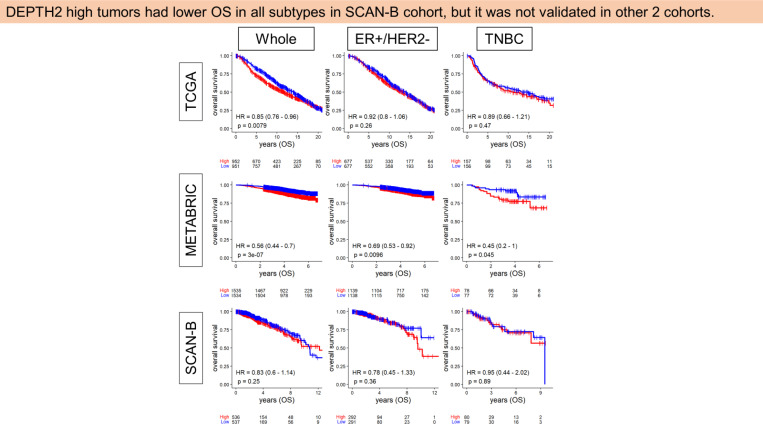
Survival outcomes in breast cancerpatients by DEPTH2 high and low groups. The Kaplan-Meier survival plots comparing between tumors with high (red lines) and low (blue lines) DEPTH2 score in TCGA, METABRIC, and SCAN-B cohorts.

Given that tumors with high ITGH are more likely to resist treatments and progress, we expected that a high DEPTH2 score which should reflect high ITGH, would be associated with worse patient survival. Our findings revealed a significant association between a high DEPTH2 score and lower OS consistently across all subtypes in the METABRIC cohort (Fig. 1; *p* < 0.05), while no such association was observed in the TCGA and SCAN-B cohorts. Higher score was associated with worse OS across all subtypes in the METABRIC cohort, but this was not confirmed in the other two cohorts, most likely due to sample size limitations (TCGA cohort showed lower OS specifically in the “whole” breast cancer subtype).

### DEPTH2 score exhibited a similar trend to ITGH calculated by ABSOLUTE, homologous recombination deficiency (HRD), silent and nonsilent mutations, as well as SNV and Indel neoantigens

**Figure 2:**
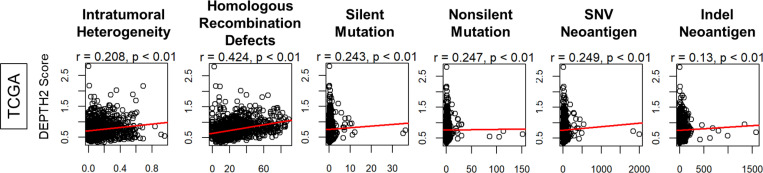
Genomic characteristics of breast cancer patients by DEPTH2 score. **(A)** Correlation plot of ITGH assessed by ABSOLUTE algorithm, as well as homologous recombination defects (HRDs), copy number alteration (CNA), silent and non-silent mutation rates, and single-nucleotide variant (SNV) and insert and deletion (Indel) neoantigens between high and low ITGH breast cancer.

It was of interest to investigate whether DEPTH2 score would be associated with other scores related to ITGH, HRDs, mutation rates, and neoantigen loads, calculated by Thorsson et al. ^29^. As shown in Fig. 2, the DEPTH2 score and all the genomic scores analyzed: ITGH by ABSOLUTE, HRDs, both silent and nonsilent mutations, and neoantigens from SNV and Indel did not show strong correlation (Fig. 2; all Pearson’s coefficient *r* < 0.5).

### High DEPTH2 tumor was associated with high cell proliferation and inversely correlated with stromal cell infiltrations

**Figure 3:**
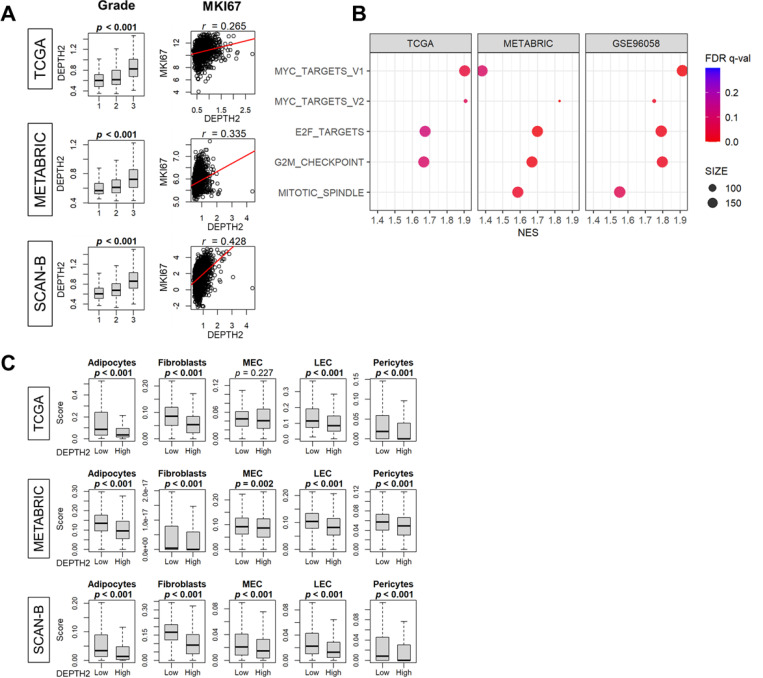
Association of DEPTH2 and cancer cell proliferation or stromal cell infiltrations in the tumor microenvironment (TME). **(A)** Association of DEPTH2 score with the Nottingham histological grade and expression of Ki-67 (MKI67), a marker of cell proliferation in TCGA, METABRIC,and SCAN-B cohorts. **(B)** Cell proliferation-related gene sets enrichment (GSEA) in breast cancer patients based on high and low DEPTH2 scores from the TCGA, METABRIC,and SCAN-B cohorts. **(C)** Analysis from TCGA, METABRIC, and SCAN-B cohorts showing infiltration levels of various stromal cell types: adipocytes, fibroblasts, microvascular endothelial cells (MEC), lymphatic endothelial cells (LEC), and pericytes.

Given that tumors with high ITGH are known to be aggressive, we investigated the relationship between the DEPTH2 score and cancer cell proliferation. We found that the DEPTH2 score correlated with Nottingham histological grade and Ki-67 gene expression (MKI67), a marker of cell proliferation, across all cohorts (Fig. 3A; all *p* < 0.001). Furthermore, tumors with high DEPTH2 scores showed enrichment in all Hallmark gene sets related to cell proliferation, including MYC Targets v1 and v2, G2M checkpoint, E2F Targets, and Mitotic Spindle. These results uniformly suggest that high DEPTH2 breast cancer is highly proliferative (Fig. 3B; all FDR < 0.25).

Following previous reports that there is an inverse relationship between cancer cell proliferation and stromal cell infiltrations in the TME^32,33^, we examined the relationship between DEPTH2 scores and stromal cell infiltrations in the TME. Our findings align with these studies, showing that breast cancers with high DEPTH2 scores were associated with decreased infiltration of adipocytes, fibroblasts, microvessel endothelial cells (MECs), lymphatic endothelial cells (LECs), and pericytes in the TME, correlating with higher cancer cell proliferation in all datasets (Fig. 3C; all p < 0.05 except for MEC in the TCGA cohort).

### Low DEPTH2 tumors enriched tumor aggravating Hallmark gene sets such as Epithelial-to-Mesenchymal Transition (EMT), Notch signaling, Angiogenesis, and Coagulation

**Figure 4:**
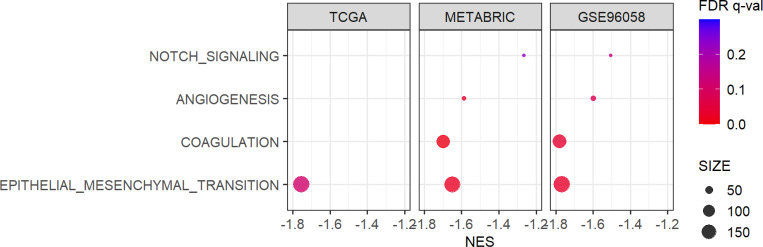
Association of DEPTH2 high and low breast cancer with Epithelial Mesenchymal Transition, COAGULATION, Angiogenesis, and NOTCH signaling gene sets. Enrichment of cancer aggravating gene sets in breast cancer by DEPTH2 score in TCGA, METABRIC, and SCAN-B cohorts.

Although tumors with high DEPTH2 scores exhibited pronounced proliferation, they were not consistently associated with worse OS across all cohorts. To this end, it was of interest to investigate whether any of the tumor aggravating pathways are associated with DEPTH2 scores. Surprisingly, multiple Hallmark gene sets linked to metastasis, including NOTCH signaling, Angiogenesis, Coagulation, and EMT, were all enriched in low, rather than high, DEPTH2 breast cancer (Fig. 4, all FDR < 0.25 except for NOTCH signaling, Angiogenesis, and Coagulation in the TCGA cohort).

#### High DEPTH2 breast cancer exhibited higher immune cell infiltrations

**Figure 5:**
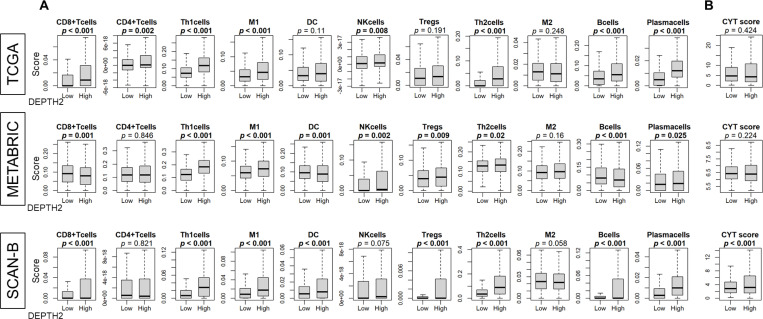
Immune cell infiltration in breast cancertumor microenvironment (TME) in relation to ITGH. **(A)** Various immune cell infiltrations in TCGA, METABRIC, and SCAN-B cohorts by DEPTH2 score high and low breast cancer groups. **(B)** Cytolytic activity (CYT) score in breast cancer cases from TCGA, METABRIC, and SCAN-B cohorts.

Previous reports indicated that high ITGH is associated with reduced immune response ^4,5^. Therefore, we investigated the relationship between the DEPTH2 score and immune cell infiltrations in three independent large cohorts. Across all three cohorts, significant infiltrations of T helper type 1 (Th1) cells, type 1 macrophages (M1), T helper type 2 (Th2) cells, and plasma cells were observed (Fig. 5, all *p* < 0.05). CD8 cells, dendritic cells (DCs), regulatory T cells (Tregs), and B cells also showed higher levels in two cohorts but were not validated in the third cohort. Additionally, cytolytic activity (CYT) indicating total immune cell-mediated destruction within the TME, was elevated in high DEPTH2 breast cancer in the SCAN-B cohort but was not significantly so in the TCGA and METABRIC cohorts (Fig. 5).

### DEPTH2 score was high in triple negative breast cancer (TNBC) subtype, but no consistent association was observed with lymph node or distal metastasis

**Figure 6:**
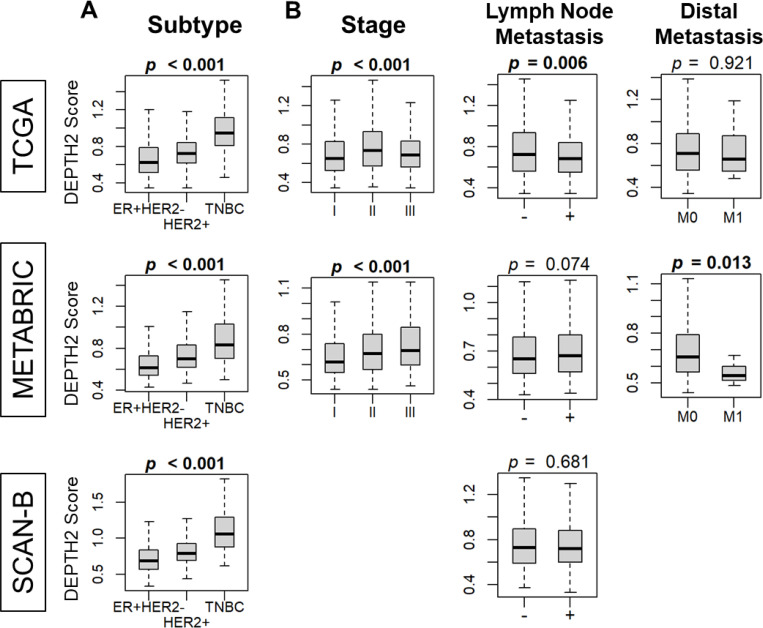
Association of high and low DEPTH2 breast cancer with clinical parameters. **(A)** Association of ITGH assessed using DEPTH2 algorithm with the breast cancer subtypes. **(B)** Relationship with the American Joint Committee on Cancer (AJCC) staging as well as lymph node and distant metastasis.

Given that a high DEPTH2 score was associated with increased cell proliferation and greater immune cell infiltration, while a low score was linked to enrichment of tumor-aggravating Hallmark gene sets, we further explored the clinical significance of the DEPTH2 score among breast cancer patients. Our results indicated that the DEPTH2 score was high in the TNBC subtype across all cohorts (Fig. 6; all *p* < 0.001). However, its relationship with the American Joint Committee on Cancer (AJCC) staging was significant in only one cohort and lacked consistency in the others (Fig. 6). Consistent with the finding that EMT gene set was enriched in low DEPTH2 breast cancer, the DEPTH2 score was associated with less lymph node metastasis in the TCGA cohort and less distal metastasis in the METABRIC cohort, but these results were not validated in the other cohort. These findings suggest that while the DEPTH2 score is associated with higher ITGH, mutation rates, neoantigens, and cell proliferation, it may not accurately reflect actual clinical parameters in breast cancer patients.

#### DEPTH2 score was not associated with response to neoadjuvant chemotherapy (NAC)

**Figure 7:**
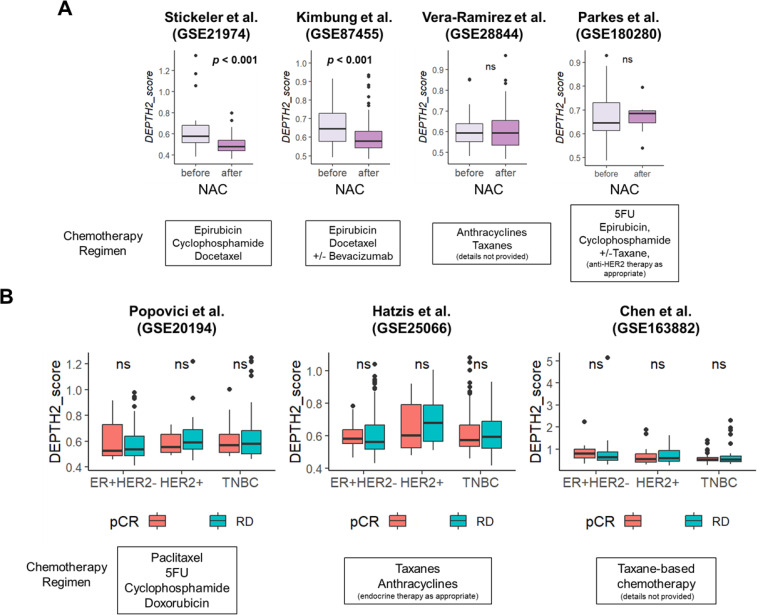
Association of chemotherapy treatment and its response in breast cancer patients by DEPTH2 high and low groups. **(A)** Association of ITGH assessed using DEPTH2 algorithm before and after neoadjuvant chemotherapy (NAC) in cohorts from Stickeler et al., Kimbung et al., Vera-Ramirez et al., and Parkes et al. **(B)** Relationship between ITGH score and pathological complete response in cohorts from Popovici et al., Hatzis et al., and Chen et al.

Given that ITGH is linked to therapeutic resistance, we investigated whether the DEPTH2 score would be associated with less response to NAC. As anticipated, the DEPTH2 score decreased after NAC in the GSE21974 and GSE87455 cohorts, but no change was observed in the GSE28844 and GSE180280 cohorts (Fig. 7A). Furthermore, the DEPTH2 score was not associated with pathologic complete response (pCR) after NAC in any of the cohorts or subtypes analyzed (Fig. 7B).

## Discussion

This study investigated the clinical relevance of DEPTH2, a computer algorithm that allows assessment of ITGH using RNA expression data alone, without the need for DNA-sequence or reference normal cell data, an improvement over the previously developed DEPTH ^12^. Through the analysis of clinicopathological and gene expression data from 6,500 breast cancer patients across seven independent cohorts, we found that breast cancer with high DEPTH2 scores was associated with lower OS consistently across all subtypes in METABRIC, but not in the TCGA and SCAN-B cohorts. The DEPTH2 score did not show strong correlation with ITGH calculated by ABSOLUTE algorithm, HRD, mutation rates, or SNV and Indel neoantigens. High DEPTH2 scores was associated with increased cancer cell proliferation, and higher immune cell infiltrations, including CD8, Th1, M1, NK cells, Tregs, Th2, and plasma cells. In contrast, low DEPTH2 tumors enriched multiple tumor aggravating Hallmark gene sets; EMT, Notch signaling, angiogenesis, and coagulation. The DEPTH2 score was significantly higher in the TNBC subtype, yet its correlation with lymph node and distal metastasis was inconsistent. Lastly, the DEPTH2 score was not associated with the response to NAC across all subtypes and the three cohorts studied.

ITGH is characterized by the coexistence of cancer cells with multiple clones that possess distinct phenotypic and molecular features within a single bulk tumor. This is prevalent in most solid human tumors including breast cancer and thought to have strong link to patient outcomes ^34–36^. ITGH is associated with treatment resistance, thus poorer prognosis. Several genome-based algorithms have been developed to date to quantify ITGH. MATH evaluates ITGH using somatic mutation profiles, focusing on the distribution of mutant-allele fractions among loci ^11^. EXPANDS characterizes coexisting subpopulations in a tumor based on copy number and allele frequencies derived from exome or whole-genome sequencing data ^10,12^. PhyloWGS infers the subclonal composition of tumor cells from mutations and CNAs, and it can be applied to whole-genome sequencing data from tumor samples to reconstruct genotypes of these subpopulations ^9^. ABSOLUTE evaluates tumor ploidy estimates representing ITGH based on DNA CNA profiles. DITHER is another algorithm evaluating ITGH using somatic mutation and CNA profiles in tumors ^8^. tITH is a transcriptome-based method for assessing ITGH which utilizes RNA-sequencing data ^13^. The concept of measuring ITGH at the spliceome level has also been reported (sITH) and represents a novel direction in ITGH evaluation ^13^; however, there are significant technical challenges in assessing sITH from bulk tumor RNA-sequence due to complex splicing patterns. DEPTH and tITH are two additional algorithms for defining ITGH at the mRNA level. DEPTH demonstrated superiority or comparability to other methods in characterizing ITGH properties than most existing algorithms ^12^.

A key advantage of DEPTH2 is its ability to assess ITGH independently of normal controls, broadening its applicability compared to DEPTH and other similar algorithms ^14^. However, accurately predicting ITGH remains challenging. Like DEPTH2, ITGH measurement is based on tissue samples that represent only a fraction of the entire tumor. Furthermore, ITGH can result not only from genomic events such as mutations, indels, and CNAs but also from epigenetic changes ^37^. Additionally, stromal and immune components significantly contribute to tumor heterogeneity and therapeutic resistance ^38^. While DEPTH2 provides valuable insights into ITGH, it does not fully demonstrate the clinical parameters and treatment responses in breast cancer patients, which we observed in the current study.

ITGH poses a significant challenge in cancer treatment, yet it is crucial for informing therapeutic choices and prognostic evaluations ^36,39^. Given that most histopathological and molecular features are not expressed homogeneously across tumor subpopulations, analysis of a single sample may lead to diagnostic and prognostic errors ^40^. This will lead to incomplete view of potential vulnerabilities to treatment ^41^. Advanced methodologies such as single-cell sequencing and spatial transcriptomics are expected to provide in-depth understanding of ITGH, but their complexity, cost, and the challenge of correlating their results with clinical outcomes limit their application in everyday clinical practice ^42^. Further, these methods still rely on very limited portion of a bulk tumor, raising concerns about whether that sample completely represents the whole tumor. In addition, the timing of ITGH evaluation during the course of treatment, and how it can be integrated with existing predictive biomarkers requires further consideration ^42^.

Although this study offers insights into the relationship between DEPTH2 score and cancer biology and patient outcomes in breast cancer, it is important to acknowledge its limitations. First, our bioinformatic results do not establish a definitive mechanism. Second, the study’s retrospective design is susceptible to selection bias. Finally, the database had incomplete clinical data, leading to the assumption that all patients received standard of care treatment.

## Conclusion

Despite being designed to characterize properties of ITGH, our findings suggest that the DEPTH2 score might not entirely represent the clinical and biological aspects of ITGH in breast cancer patients. Additional research is required to develop an effective computational algorithm to assess ITGH with RNA expression data without requiring DNA-sequencing or reference normal cell data.

## Data Availability

The datasets analyzed during the current study are available in the cBioPortal (https://www.cbioportal.org) and Gene Expression Omnibus (GEO) repository of the US National Institutes of Health (https://www.ncbi.nlm.nih.gov/geo).
